# Supplementary material for: Recovering true FRET efficiencies from smFRET investigations requires triplet state mitigation
Source: Nat Methods. 2024 Jun 14;21(7):1222–30. doi: 10.1038/s41592-024-02293-8 (PMC11239528; doi:10.1038/s41592-024-02293-8)
Supplement: Supplementary file 1 — Supplementary Figs. 1–18, Supplementary Tables 1–6 and Supplementary Schemes 1 and 2. [file 41592_2024_2293_MOESM1_ESM.pdf]

nature methods

Supplementary information https://doi.org/10.1038/s41592-024-02293-8

Recovering true FRET efficiencies from smFRET investigations requires triplet state mitigation

In the format provided by the authors and unedited

Table of contents:

**Supplementary Fig. 1**	Molecular structures of FRET dye pairs
**Supplementary Fig. 2**	smFRET imaging of Cy3-Cy5 in oxygenated buffers in the absence of additives
**Supplementary Fig. 3**	smFRET studies of Cy3-Cy5 and LD555-LD655 using confocal illumination strategies
**Supplementary Fig. 4**	Illumination intensity-dependent smFRET efficiency
**Supplementary Fig. 5**	smFRET imaging of Cy3-Cy5 in deoxygenated buffers in the presence of additives
**Supplementary Fig. 6**	smFRET imaging of Cy3-Cy5 in oxygenated buffers in the presence of additives
**Supplementary Fig. 7**	smFRET imaging of ATTO550-ATTO647N in deoxygenated and oxygenated buffers
**Supplementary Fig. 8**	Fluorescence correlation spectroscopy studies of Cy3B and Cy5B
**Supplementary Fig. 9**	smFRET imaging of LD555-LD655 in oxygenated buffers in the absence of additives
**Supplementary Fig. 10**	Illumination intensity-dependent FRET efficiency of Cy3-LD655 and LD555-Cy5
**Supplementary Fig. 11**	Photon count rate of Cy3B at various illumination intensities
**Supplementary Fig. 12**	Illumination intensity-dependent FRET efficiency of Cy3-Cy5 and LD555-LD655 at various ISC rates and triplet lifetimes and empirical γ correction
**Supplementary Fig. 13**	Simulation study of FRET efficiency of Cy3-Cy5 and LD555-LD655 at various donor-acceptor distances
**Supplementary Fig. 14**	Existing and proposed correction methods for FRET efficiencies.
**Supplementary Fig. 15**	Summary of contour plots provided in Supplementary Fig. 14
**Supplementary Fig. 16**	ζ correction parameters of Cy3-Cy5 and LD555-LD655 considering various excited state processes
**Supplementary Fig. 17**	High spatial resolution FRET study of Cy3-Cy5 and ATTO550-ATTO647N
**Supplementary Fig. 18**	High spatial resolution FRET study of LD555-LD655
**Supplementary Table 1**	Photophysical parameters of FRET dyes.
**Supplementary Table 2**	Förster radius for the dye pairs used in Fig. 2
**Supplementary Table 3**	Description of theoretical FRET efficiency for 4 and 9 state models
**Supplementary Table 4**	Description of simulated and experimental FRET efficiencies
**Supplementary Table 5**	Description of theoretical FRET efficiencies from $E_{theory}^{4\,st}$
**Supplementary Table 6**	Description of theoretical FRET efficiencies from $E_{theory}^{9\,st}$
**Supplementary Scheme 1**	A 4-state photophysical model
**Supplementary Scheme 2**	A 9-state photophysical model

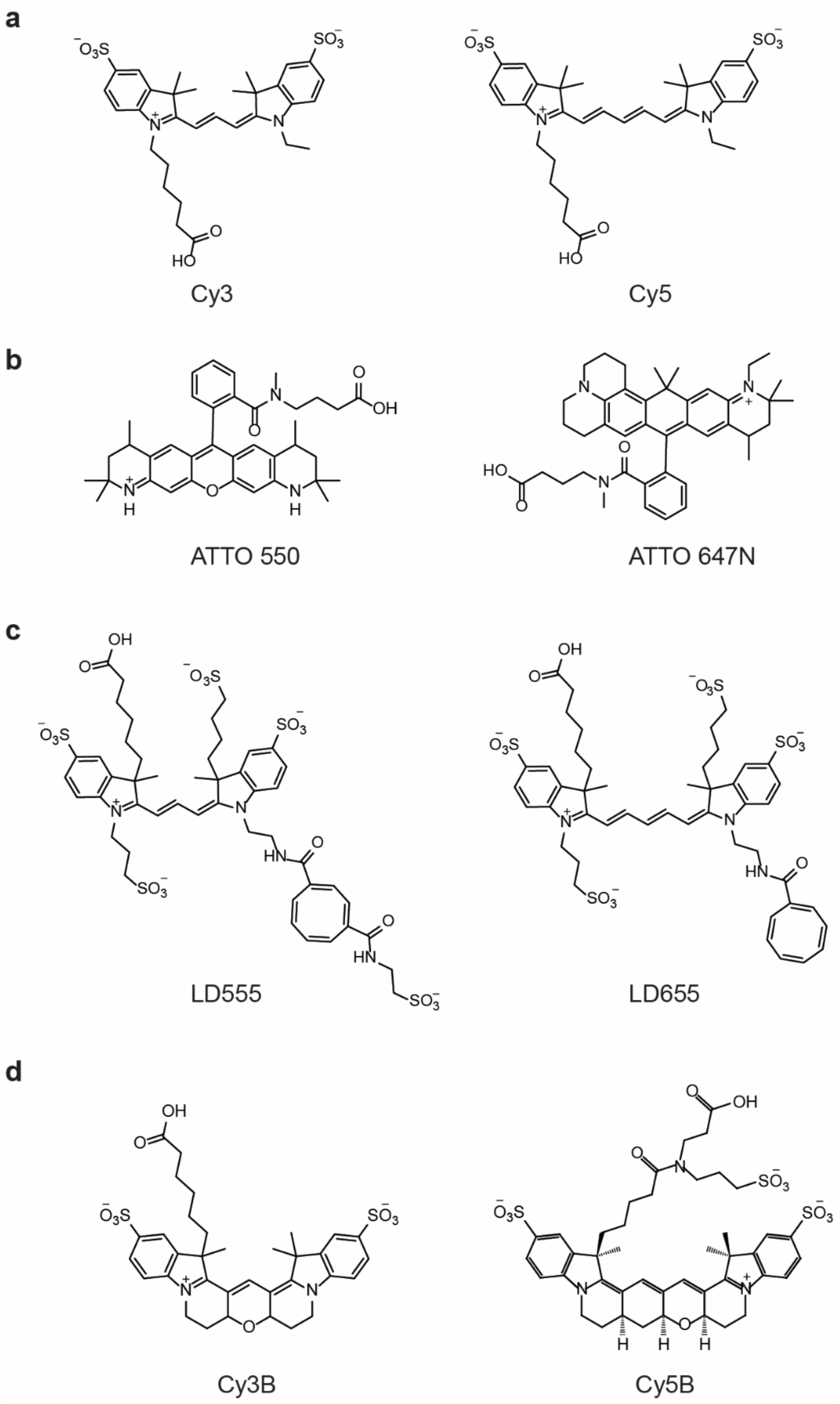

Supplementary Fig. 1. Molecular structures of FRET dye pairs. Structures of (**a**) Cy3, Cy5; (**b**) ATTO550, ATTO647N; (**c**) LD555, LD655; and (**d**) Cy3B, Cy5B fluorophores.

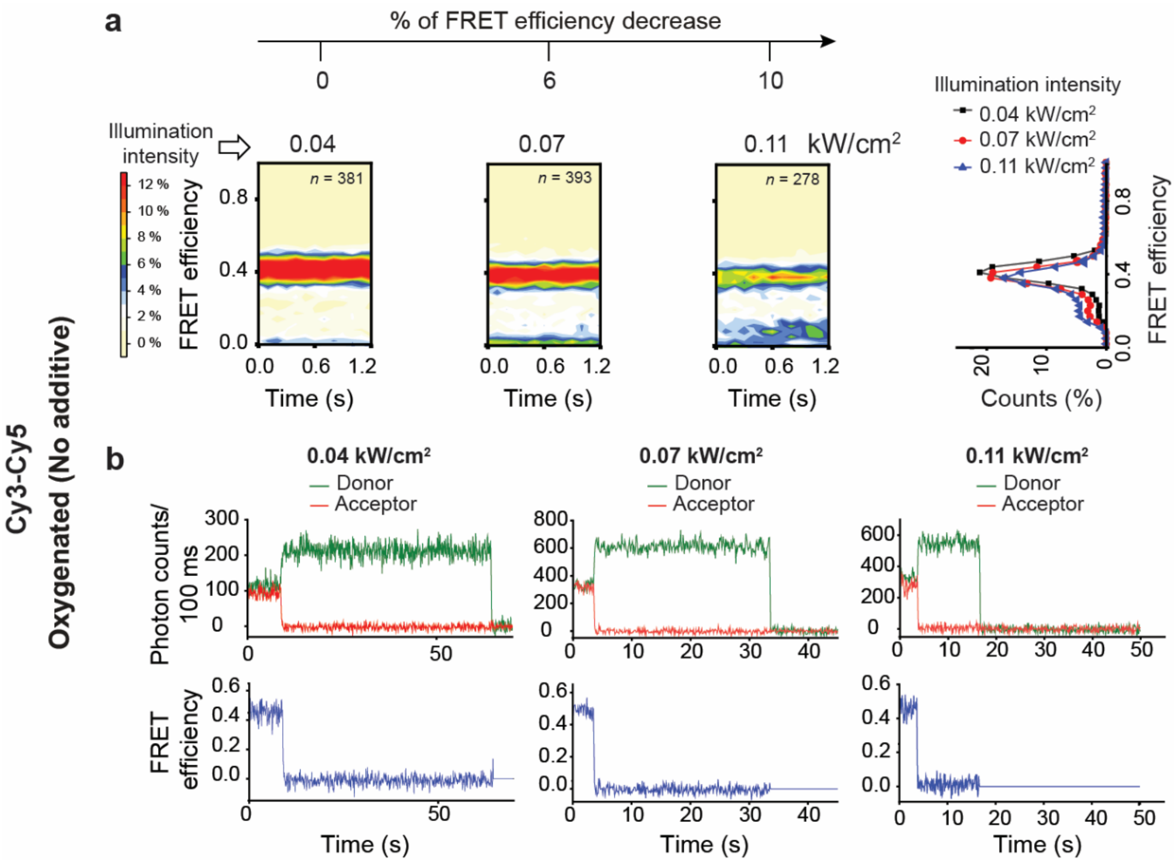

Supplementary Fig. 2. smFRET imaging of Cy3-Cy5 in oxygenated buffers in the absence of additives. (**a**) Population FRET efficiency contour plots (cumulative population FRET efficiency histograms on the right) and (**b**) single-molecule fluorescence (top row) and FRET efficiency (bottom row) traces of Cy3-Cy5 pair attached to a DNA duplex (**Fig. 2a**) in oxygenated imaging buffers in the absence of solution additives at 100 ms time resolution. The number of single molecules was relatively low due to fast photobleaching in the presence of oxygen. The % of FRET efficiency decrease was calculated with respect to the lowest power tested (0.04 kW/cm^2).

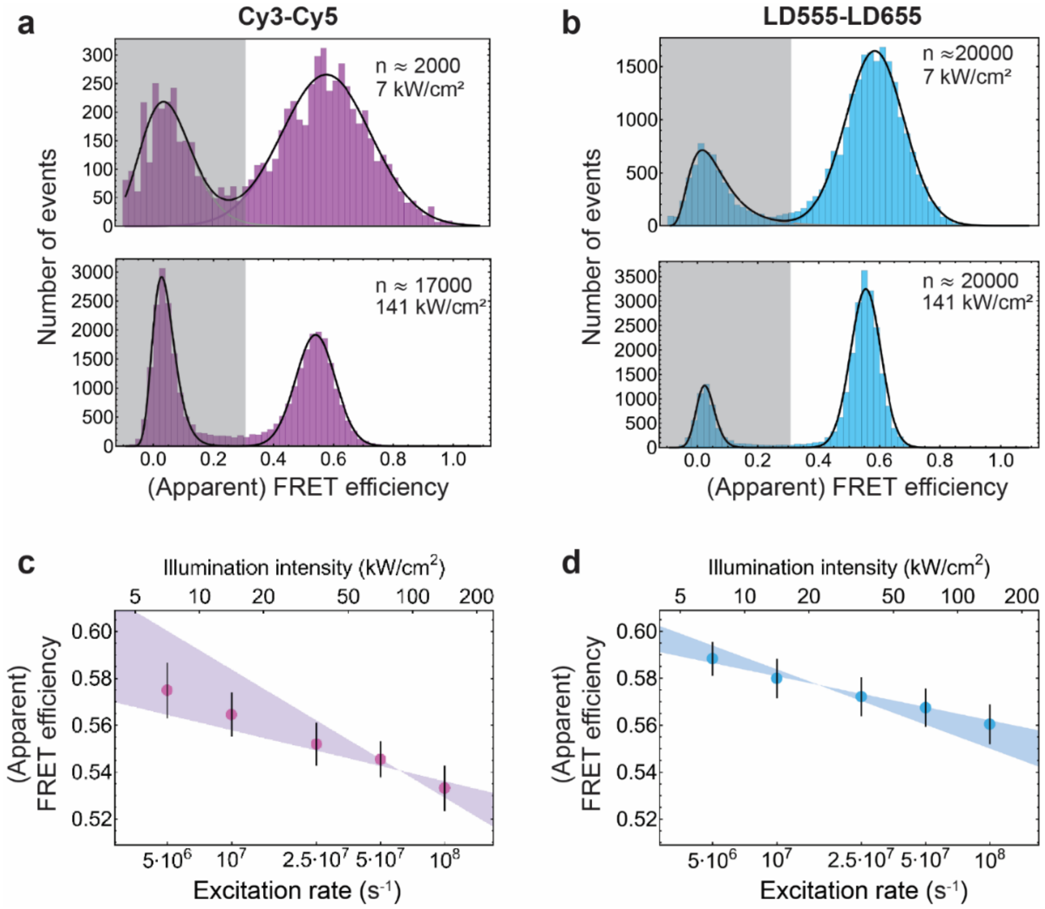

Supplementary Fig. 3. smFRET studies of Cy3-Cy5 and LD555-LD655 using confocal illumination. Apparent FRET efficiency histograms of DNA duplex (**Fig. 2a**) labeled with (**a**) Cy3-Cy5 and (**b**) LD555-LD655 FRET pair recorded in a confocal setup as a function of illumination intensity in deoxygenated conditions and in the absence of triplet state quenchers (illumination intensity and sample size in insets). Fluorescence intensities were corrected for background and direct excitation but not for crosstalk and histograms were fitted with Gaussian peak functions. For both dye pairs, the gray shaded area covers a population of molecules without an active acceptor dye. Insets indicate the approximate number of molecules recorded for the histograms shown (counting only those with FRET efficiency ≥ 0.3). (**c,d**). Plot of the apparent FRET efficiency of dsDNA constructs labeled with (**c**) Cy3-Cy5 and (**d**) LD555-LD655 FRET pair as a function of excitation power, here expressed as both illumination intensity (top x axis) and donor fluorophore excitation rate k_{ex}^{D} (bottom x axis), calculated from the values reported in insets (**Methods**). The magenta and blue circles are the average of two independent measurement series; vertical bars through circles are the standard deviation of two independent measurements. Violet and dark blue shaded areas are delimited by linear fits of the apparent FRET efficiency values obtained by varying the inter-photon time threshold used for bursts selection (**Methods**): a 30% longer time yields the line with lower negative slope, while a 30% shorter time returns the higher negative-slope line in both cases.

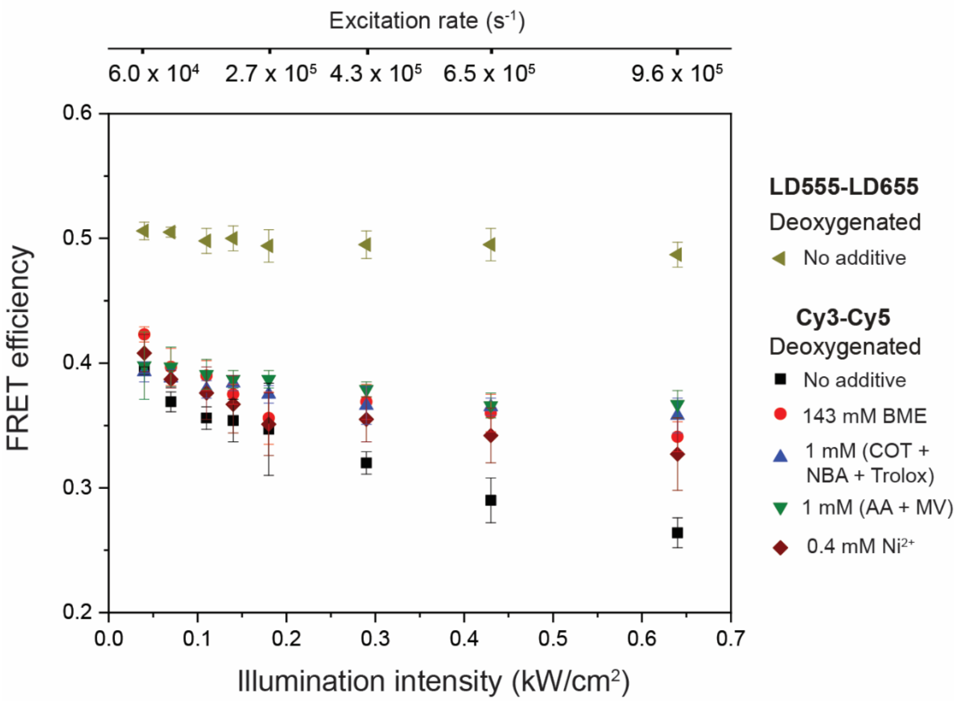

Supplementary Fig. 4. Illumination intensity-dependent smFRET efficiency. Variation of FRET efficiency with increasing illumination intensities of Cy3-Cy5 and LD555-LD655 FRET pairs attached to a DNA duplex (**Fig. 2a**) in deoxygenated imaging buffers in the absence and presence of solution additives at 100 ms time resolution. COT, NBA, AA, MV, BME represent cyclooctatetraene, 4-nitrobenzyl alcohol, ascorbic acid, methyl viologen, *β*-mercaptoethanol, respectively. The error bars are the standard deviation of mean FRET efficiency values from five experimental repeats.

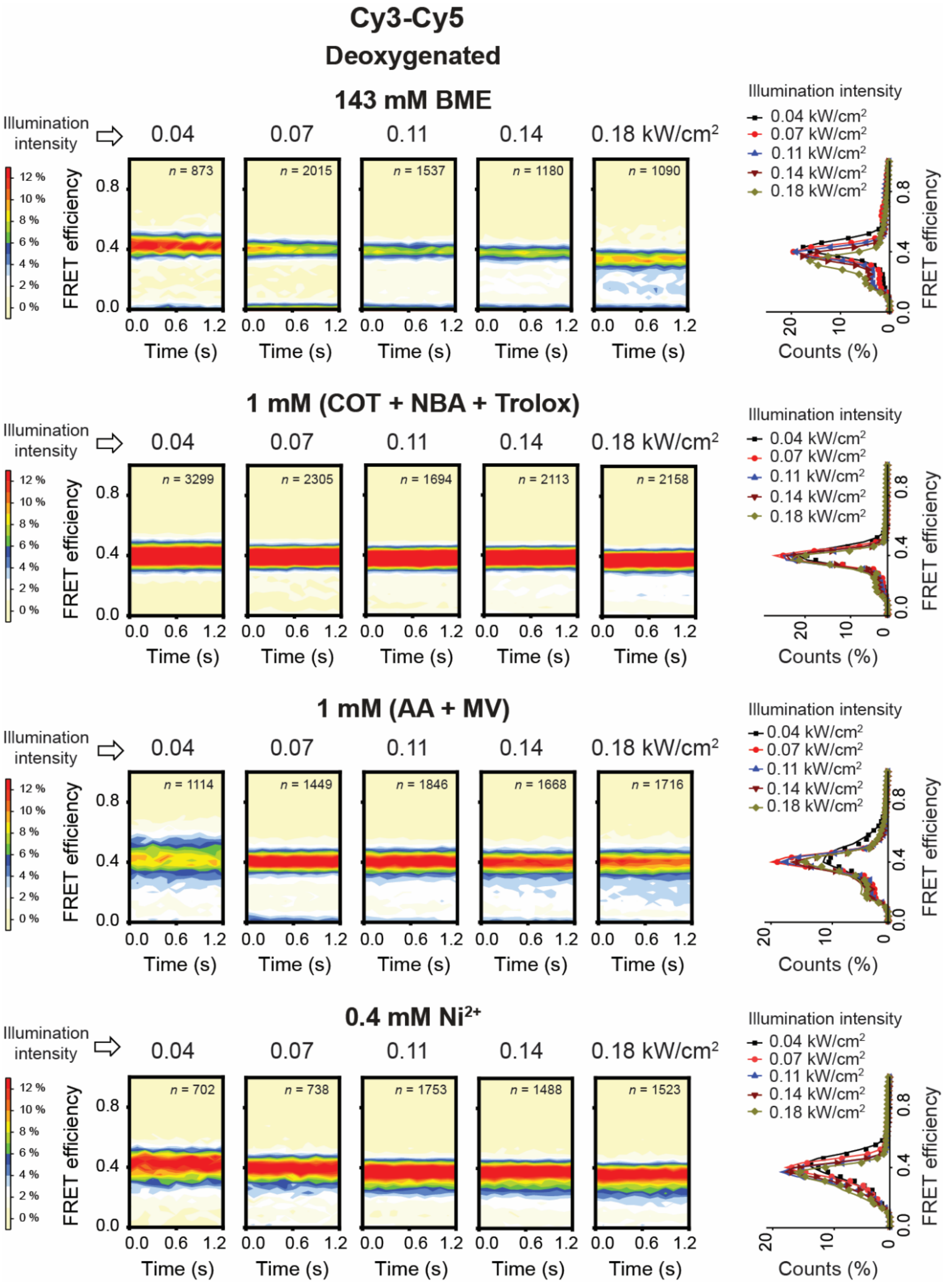

Supplementary Fig. 5. smFRET imaging of Cy3-Cy5 in deoxygenated buffers in the presence of additives. Population FRET efficiency contour plots of the Cy3-Cy5 pair attached to a DNA duplex (**Fig.**

2a) in deoxygenated imaging buffers in the presence of solution additives at 100 ms time resolution. Cumulative population FRET efficiency histograms are shown on the right. COT, NBA, AA, MV, BME represent cyclooctatetraene, 4-nitrobenzyl alcohol, ascorbic acid, methyl viologen, *β*-mercaptoethanol, respectively. The imaging data in the absence of any additives are depicted in **Fig. 2c,d**.

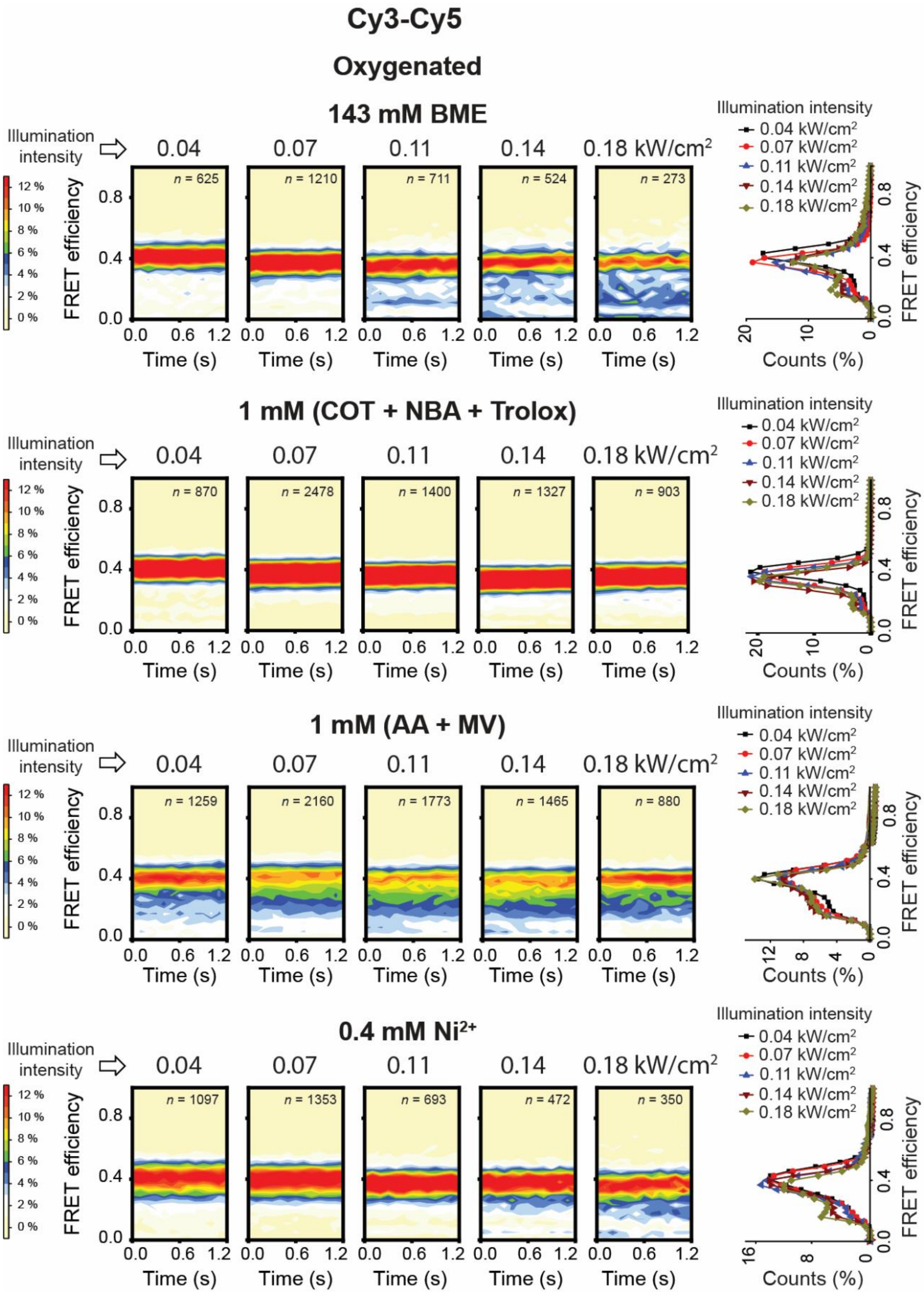

Supplementary Fig. 6. smFRET imaging of Cy3-Cy5 in oxygenated buffers in the presence of additives. Population FRET efficiency contour plots of the Cy3-Cy5 pair attached to a DNA duplex (**Fig.**

2a) in oxygenated imaging buffers in the presence of solution additives at 100 ms time resolution. Cumulative population FRET efficiency histograms are shown on the right. COT, NBA, AA, MV, BME represent cyclooctatetraene, 4-nitrobenzyl alcohol, ascorbic acid, methyl viologen, *β*-mercaptoethanol, respectively. The imaging data in the absence of any additives are depicted in **Supplementary Fig. 2**.

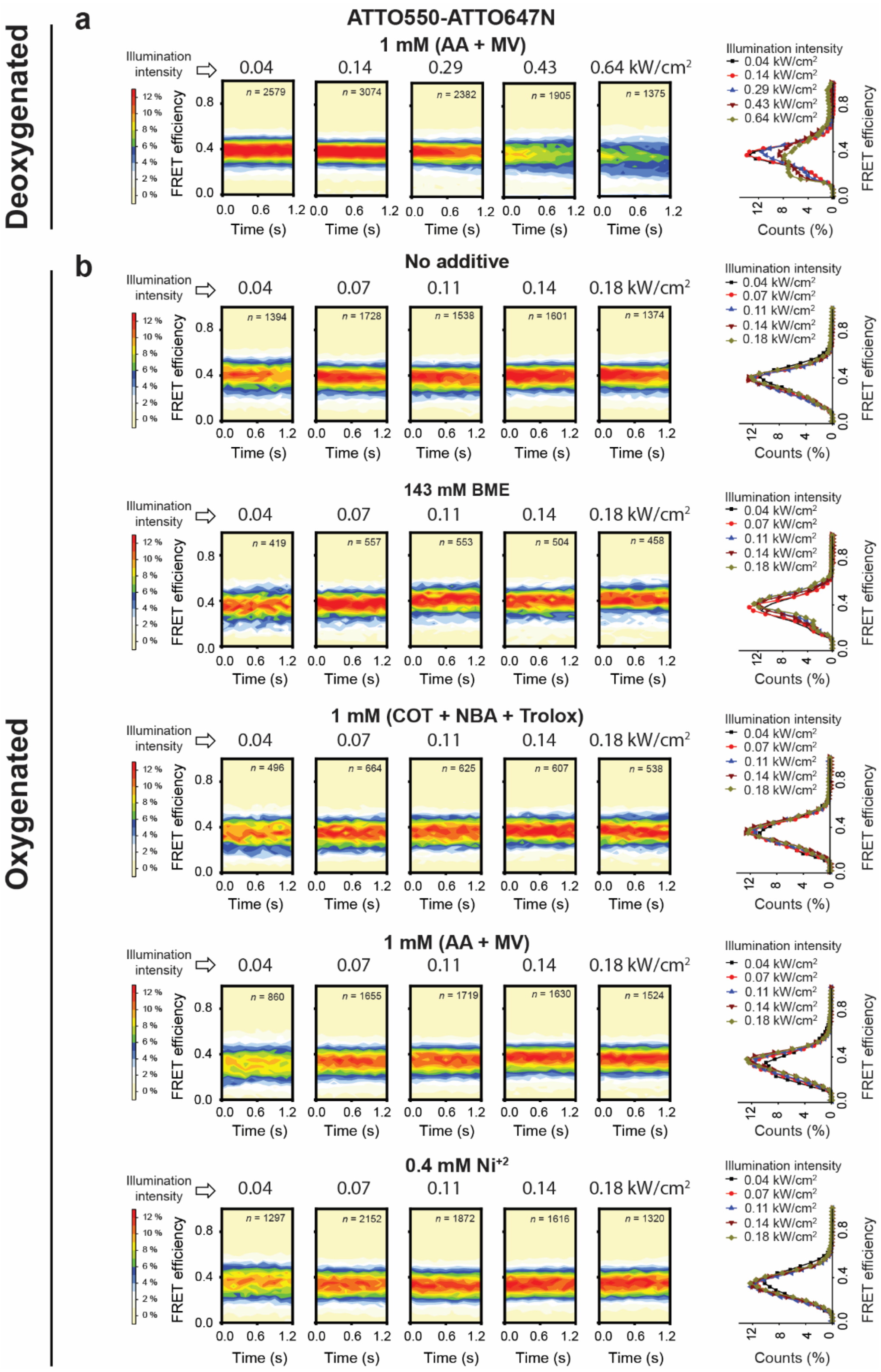

Supplementary Fig. 7. smFRET imaging of ATTO550-ATTO647N in deoxygenated and oxygenated buffers. Population FRET efficiency contour plots of the ATTO 550-ATTO 647N pair attached to a DNA

duplex (**Fig. 2a**) in (**a**) deoxygenated and (**b**) oxygenated imaging environments in the absence and presence of various solution-based photostabilizers at 100 ms time resolution. Cumulative population FRET efficiency histograms are shown on the right. COT, NBA, AA, MV, BME represent cyclooctatetraene, 4-nitrobenzyl alcohol, ascorbic acid, methyl viologen, β-mercaptoethanol, respectively.

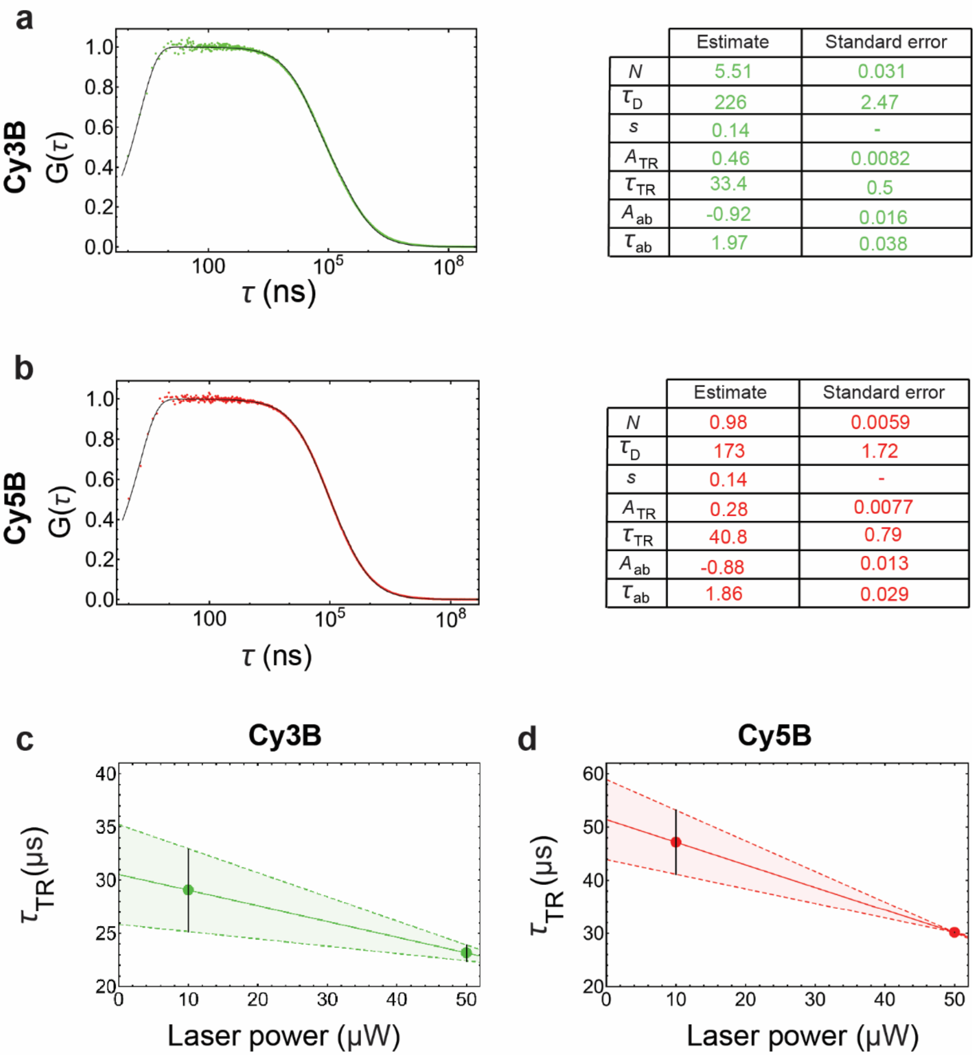

	Estimate	Standard error
N	5.51	0.031
τ_D	226	2.47
s	0.14	-
A_{TR}	0.46	0.0082
τ_{TR}	33.4	0.5
A_{ab}	-0.92	0.016
τ_{ab}	1.97	0.038

	Estimate	Standard error
N	0.98	0.0059
τ_D	173	1.72
s	0.14	-
A_{TR}	0.28	0.0077
τ_{TR}	40.8	0.79
A_{ab}	-0.88	0.013
τ_{ab}	1.86	0.029

Supplementary Fig. 8. Fluorescence correlation spectroscopy studies of Cy3B and Cy5B. Fluorescence correlation curves of (**a**) Cy3B and (**b**) Cy5B for the estimation of triplet lifetimes, recorded in a confocal setup in deoxygenated buffer (**Methods**) and in the absence of solution additives. The correlations were fitted with a model including antibunching, triplet blinking, and diffusion (**Methods**). The right-side tables contain fitting parameters for the correlations shown: *N* is the average number of molecules in the confocal volume, τ_{D} is the diffusion time in microseconds (μs), *s* is the ratio of the lateral to the axial radii of the confocal volume; A_{TR} and τ_{TR} are the amplitude and lifetime (in μs) of the triplet state population; τ_{ab} is the antibunching time in ns and A_{ab} is its associated amplitude**.** These rigidified Cy3 (Cy3B) and Cy5 (Cy5B) dyes simplify the fluorescence correlation curves so that triplet state lifetimes can be recovered more robustly. Average triplet lifetimes were obtained from two and three independent measurements at 50 and 10 μW (corresponding to 35 and 7 kW/cm^2), respectively, for Cy3B (**c**, green circled), and from 3 independent measurements at 10 μW for Cy5B (**d**, red circles); vertical bars through circles are the standard deviation. The mean triplet lifetime and associated uncertainty were then linearly extrapolated to zero power (solid and dashed lines, respectively, in both cases), yielding more accurate values of 31 ± 5 μs and 51 ± 8 μs for the triplet lifetimes of Cy3B and Cy5B, respectively.

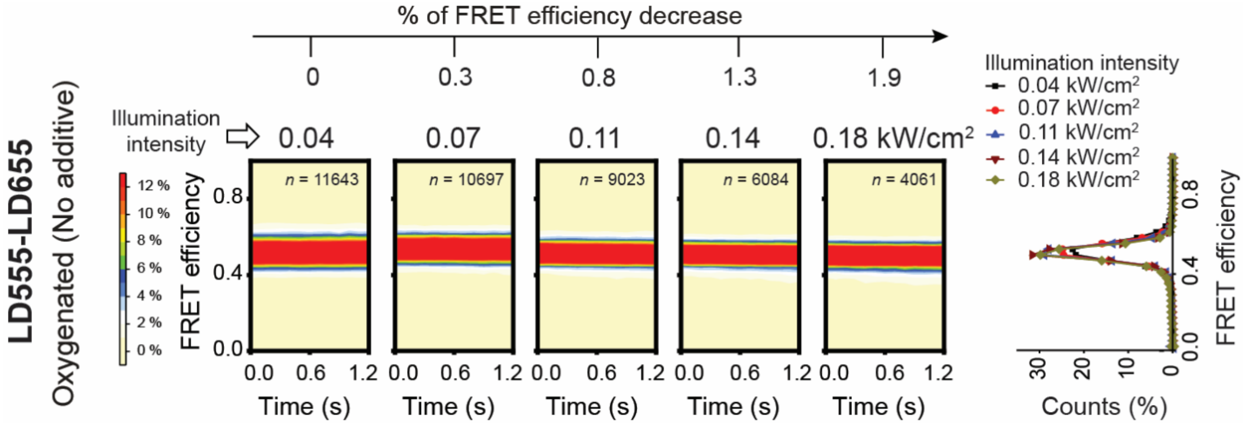

Supplementary Fig. 9. smFRET imaging of LD555-LD655 in oxygenated buffers in the absence of additives. Population FRET efficiency contour plots of the LD555-LD655 pair attached to a DNA duplex (**Fig. 2a**) in oxygenated imaging environments in the absence of solution additives at 100 ms time resolution. Cumulative population FRET efficiency histograms are shown on the right. The % of FRET efficiency decrease was calculated with respect to the lowest power tested (0.04 kW/cm^2).

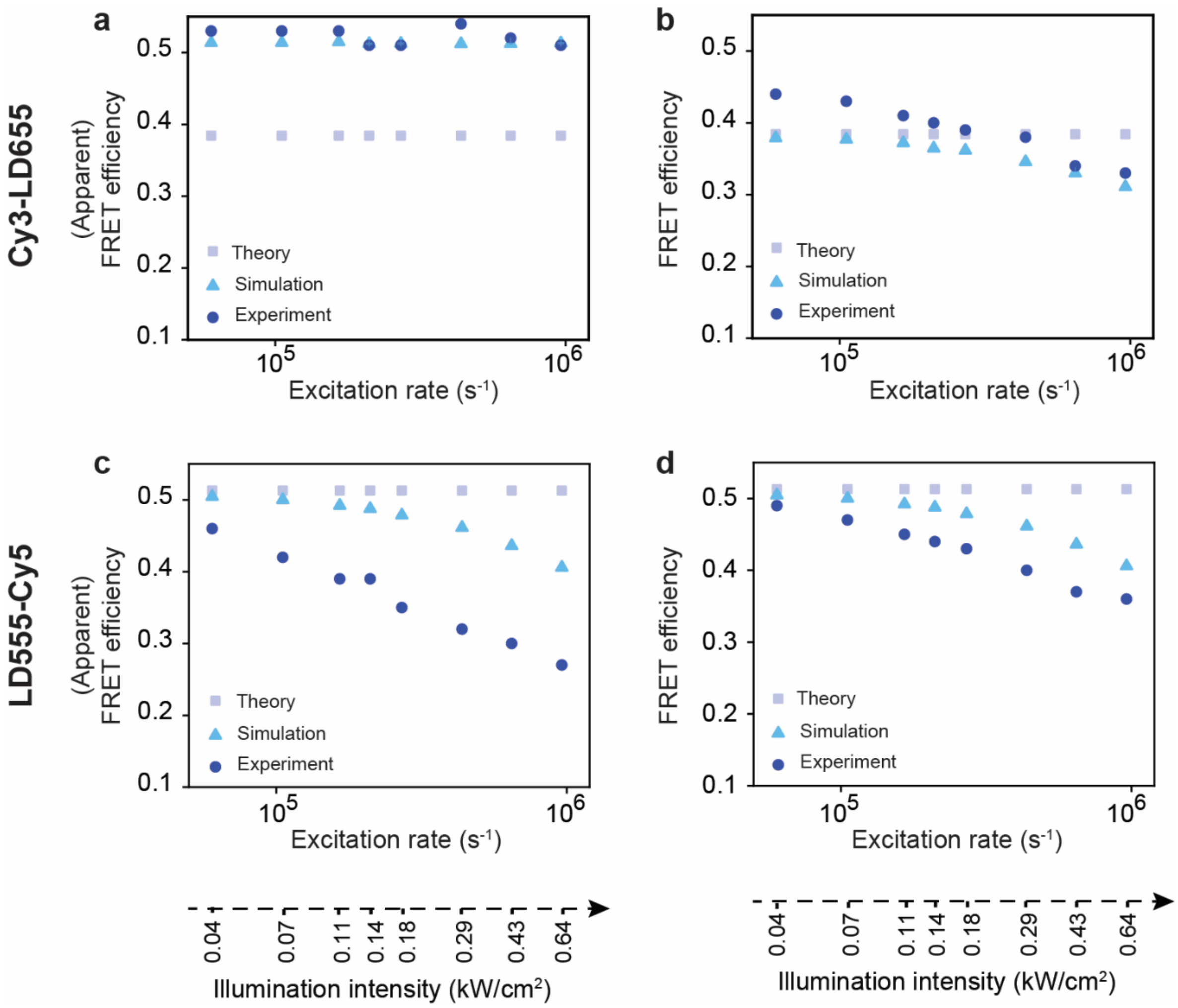

Supplementary Fig. 10. Illumination intensity-dependent FRET efficiency of Cy3-LD655 and LD555-Cy5. Variations of (**a**,**c**) apparent FRET efficiency and (**b**,**d**) FRET efficiency of Cy3-LD655 (**a**,**b**) and LD555-Cy5 (**c**,**d**) pairs attached to a DNA duplex (**Fig. 2a**) with increasing excitation rates. The photophysical parameters of the dyes used in the simulations are tabulated in **Supplementary Tables 1,2**. The theoretical FRET efficiency was calculated with $E_{theory}^{9\,st}$ (**Supplementary Table 3**); the apparent FRET efficiency was calculated without γ-correction factor; and the FRET efficiency calculation included the γ correction factor (**Methods**). The experimental data were collected in deoxygenated imaging buffers at 100 ms time resolution in the absence of solution additive.

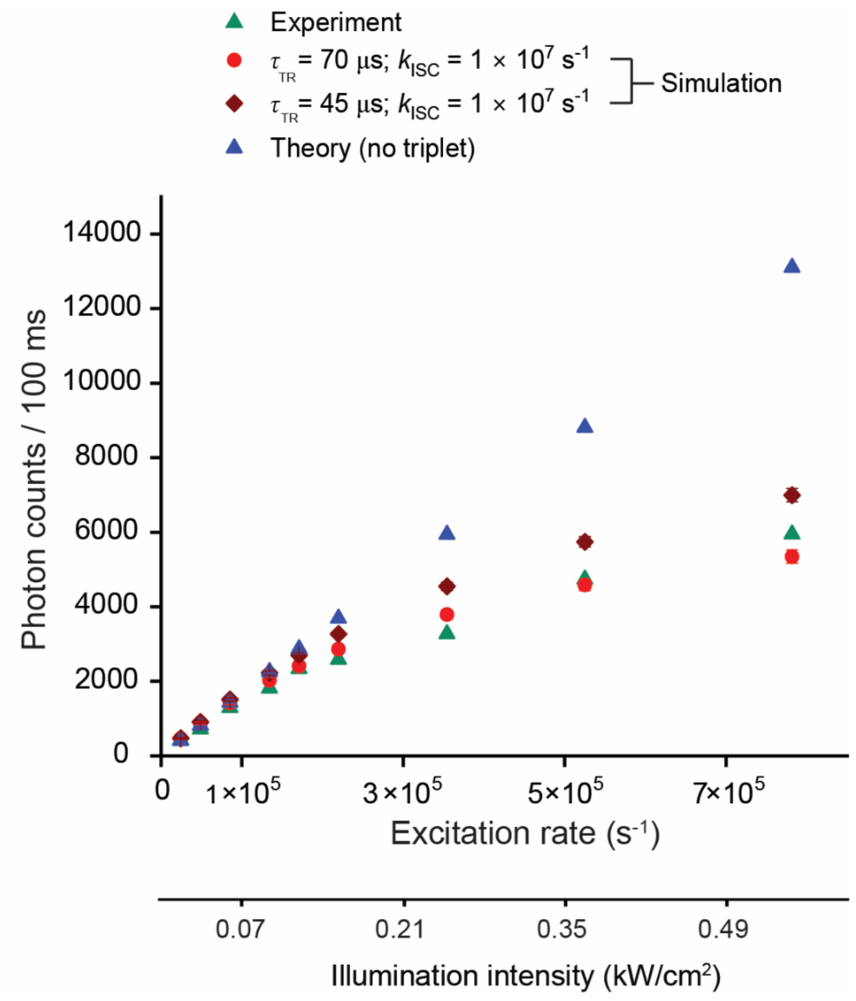

Supplementary Fig. 11. Photon count rate of Cy3B at various illumination intensities. Simulations of photon counts of Cy3B at various excitation rates. The simulations were performed by considering photophysical parameters of a Cy3B in the absence of an acceptor while attached to a DNA duplex; **Supplementary Table 1**). The simulated data were compared with experimental imaging data collected in deoxygenated imaging buffers at 100 ms time resolution in the absence of any solution additive. τ_{TR} and k_{ISC} indicate the triplet state lifetime and intersystem crossing rate constant, respectively.

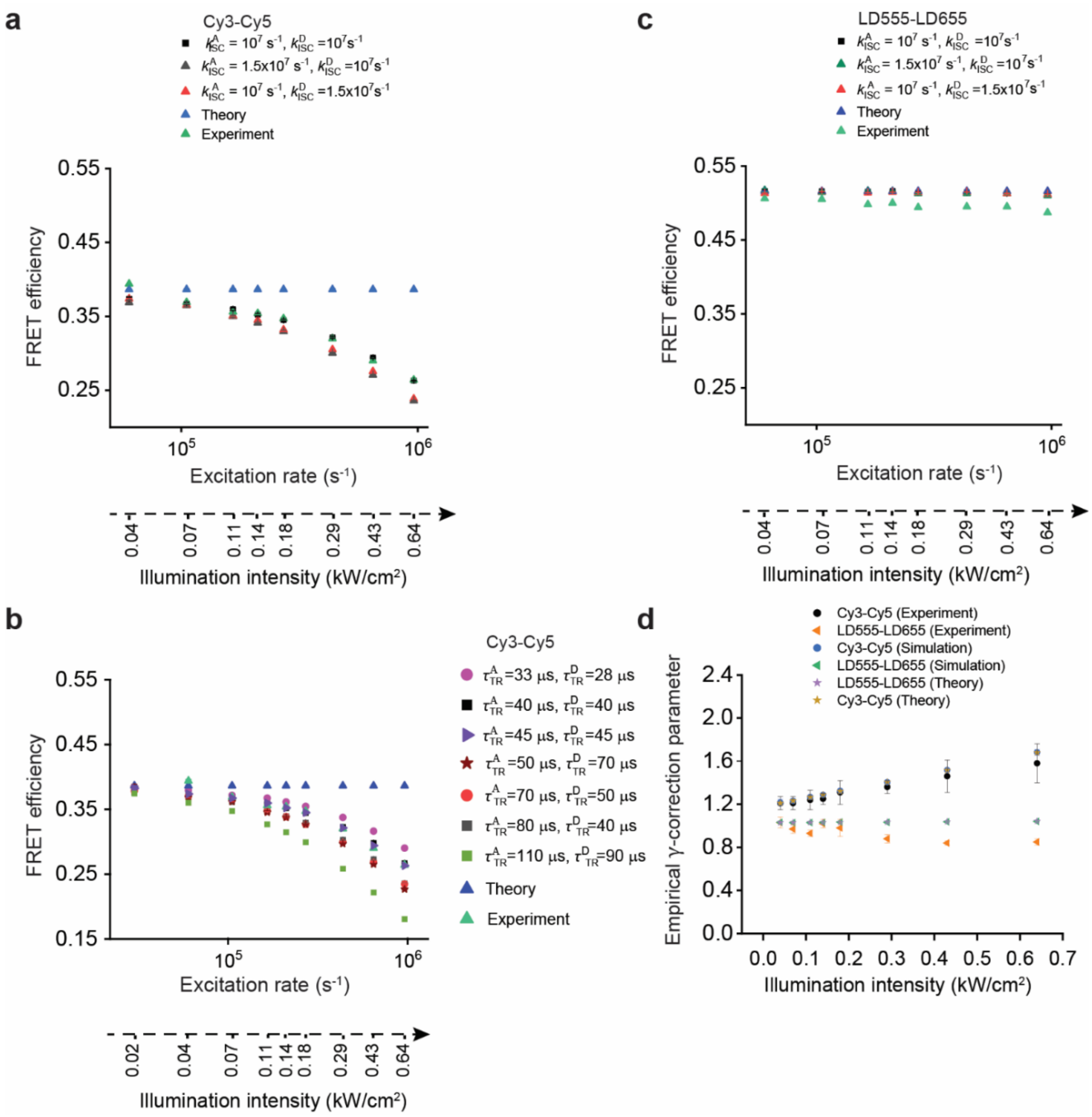

Supplementary Fig. 12. Illumination intensity-dependent FRET efficiency of Cy3-Cy5 and LD555-LD655 at various ISC rates and triplet lifetimes and empirical γ correction. (**a**,**c**) Simulations of FRET efficiency of (**a**) Cy3-Cy5 and (**c**) LD555-LD655 pairs with increasing excitation rates at various simulated intersystem crossing rates. (**b**) Simulations of FRET efficiency of the Cy3-Cy5 pair with increasing excitation rates including donor and acceptor triplet state lifetimes. The photophysical parameters of the dyes used in the simulations are tabulated in **Supplementary Table 1**. The theoretical FRET efficiency was calculated without considering any triplet state ($E_{theory}^{9\,st}$, **Supplementary Table 3**). The experimental data were collected in deoxygenated imaging environment at 100 ms time resolution in the absence of any solution additive. k_{ISC}^{D}, k_{ISC}^{A}, τ_{TR}^{D} and τ_{TR}^{A} indicate donor and acceptor dye intersystem crossing rate constants, and their corresponding triplet relaxation lifetimes, respectively. (**d**) Variations of experimental, theoretical, and simulated empirical γ correction parameters for a set of FRET pairs across illumination intensities. We match the experimental and simulated empirical γ corrections at the first illumination intensity by multiplying simulated empirical γ corrections for Cy3-Cy5 and LD555-LD655 data with 0.802 and 0.913, respectively. Error bars for Cy3-Cy5 and LD555-LD655 experimental data represent the standard deviation of mean γ values from five experimental repeats.

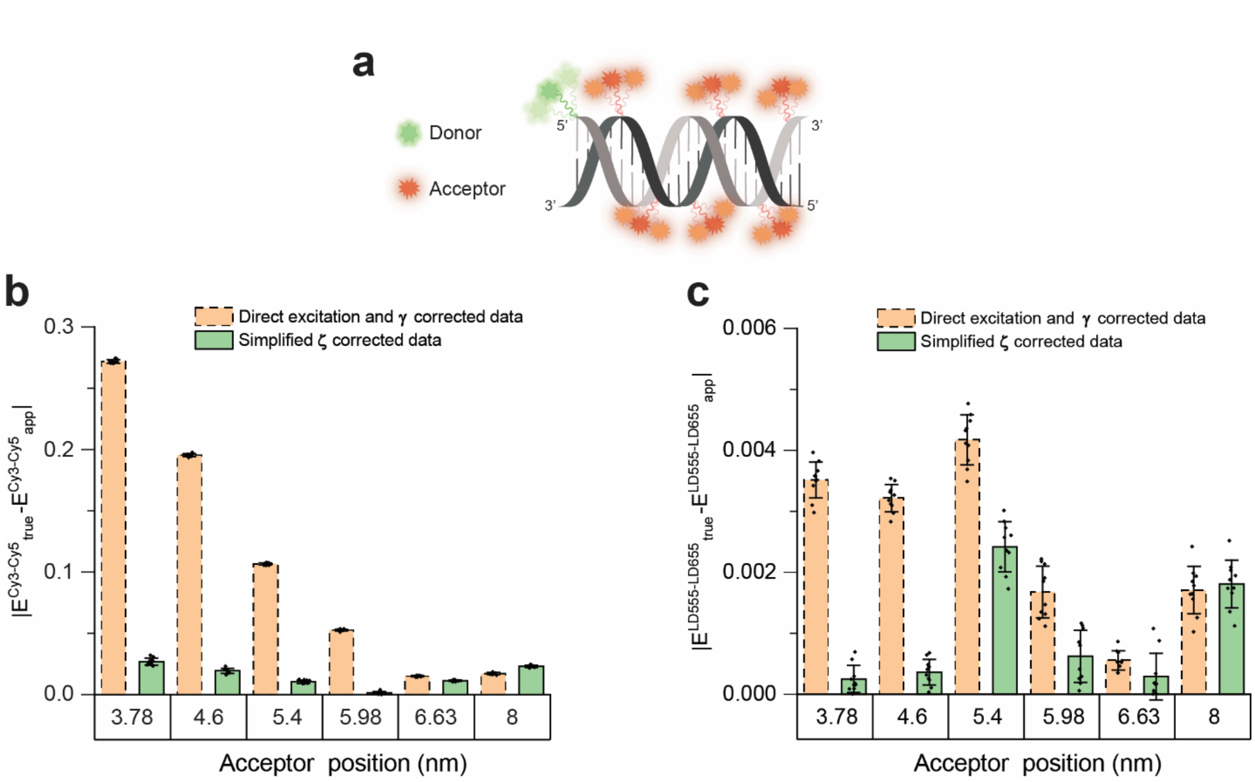

Supplementary Fig. 13. Simulation study of FRET efficiency of Cy3-Cy5 and LD555-LD655 at various donor-acceptor distances. (**a**) Schematic diagram of a mixture of six DNA duplexes labeled with donor fluorophore at the 5'-terminus of one strand and acceptor fluorophore positioned at internal sites within the complementary strand separated by 5, 8, 11, 14, 17, and 20 nucleotides. (**b**,**c**) Distance-dependent changes in FRET efficiency due to triplet state accumulations for the (**b**) Cy3-Cy5 and (**c**) LD555-LD655 FRET pairs. Tan (dashed boarder) represents raw simulated data in the absence of cross-talk that has been corrected by standard practice procedures using theoretical γ correction (applied to the whole simulation across all FRET efficiencies); green (solid boarder) represents the same data, ζ-corrected with the simplified 9-state photophysical model in the absence of direct excitation or distance considerations. Mean FRET efficiencies are obtained from the individual simulated FRET trajectories across all distances. Subsequently, the mean FRET efficiencies are corrected with the simplified (green) ζ corrections. Error bars represent the standard deviation for the FRET efficiency differences from simulation of 10 seconds long 10 single-molecule trajectories. Simulations were carried out at an excitation rate (k_{ex}^{D}) of ~10^6 s^{-1}, a power that represents the high-end of TIRFM and low-end of confocal illumination intensities employed at 100 ms time resolution.

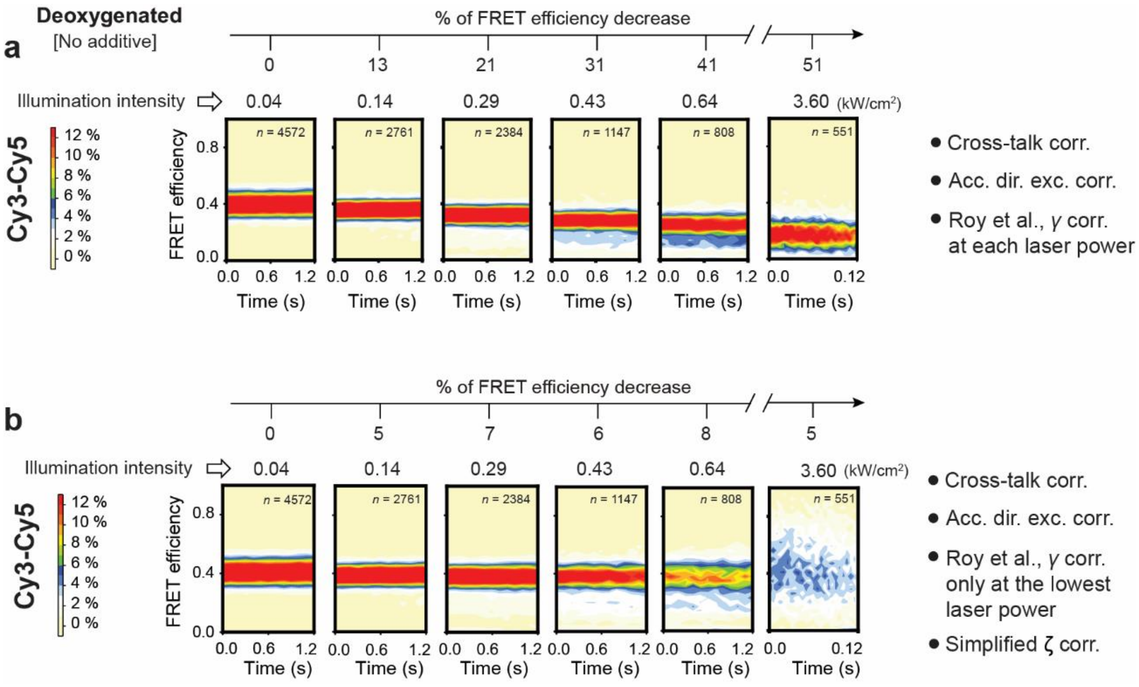

Supplementary Fig. 14. Existing and proposed correction methods for FRET efficiencies. (**a**) Corrections of Cy3-Cy5 FRET efficiencies using standard -practice procedures[1–3] at various illumination intensities. Data in panel (**a**) are the same as shown in main text **Fig. 2c** (top row, panel a). (**b**) Simplified (9-state model) ζ-correction of the data shown in panel a, where the corrections before simplified ζ-correction are presented on the right most panel in the same row. Determination of the percentage decrease in FRET efficiency is normalized relative to the mean FRET efficiency obtained at the lowest illumination intensity.

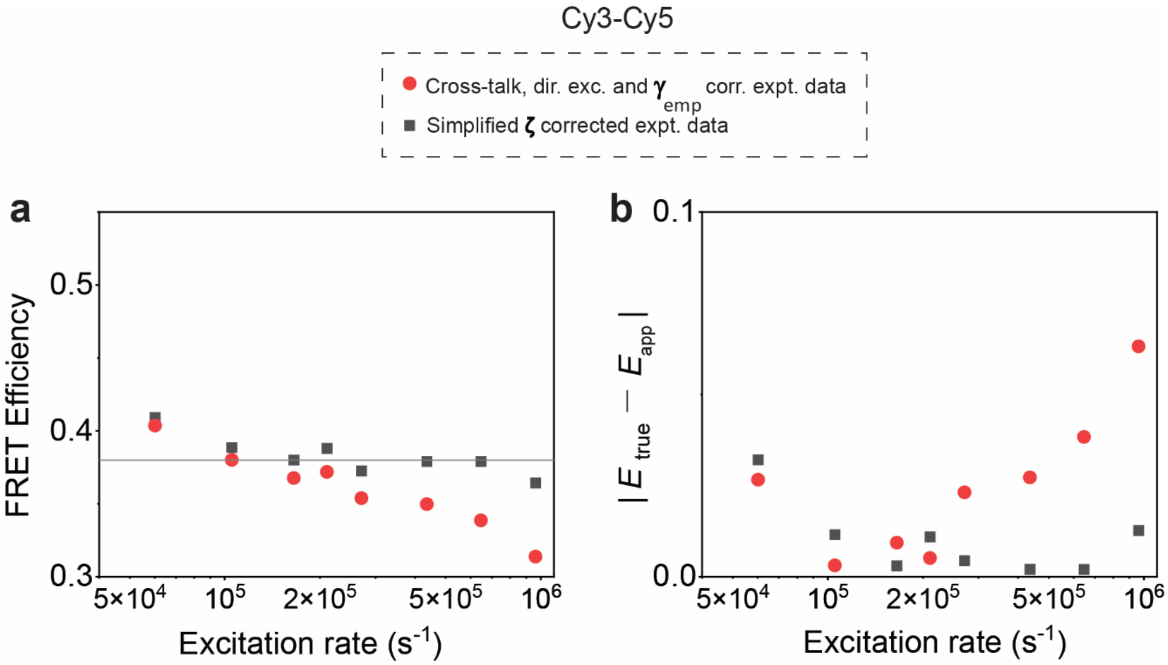

Supplementary Fig. 15. Summary of contour plots provided in Supplementary Fig. 14. (**a**) Variation of mean FRET efficiency of Cy3-Cy5 FRET pair corrected using existing standard and proposed approaches with excitation rates and (**b**) their net changes from the true FRET efficiency (shown in gray reference line) value obtained with $\frac{1}{1+(\frac{R}{R_0})^6}$ where $R = 5.98\ nm$ and $R_0 = 5.5\ nm$.

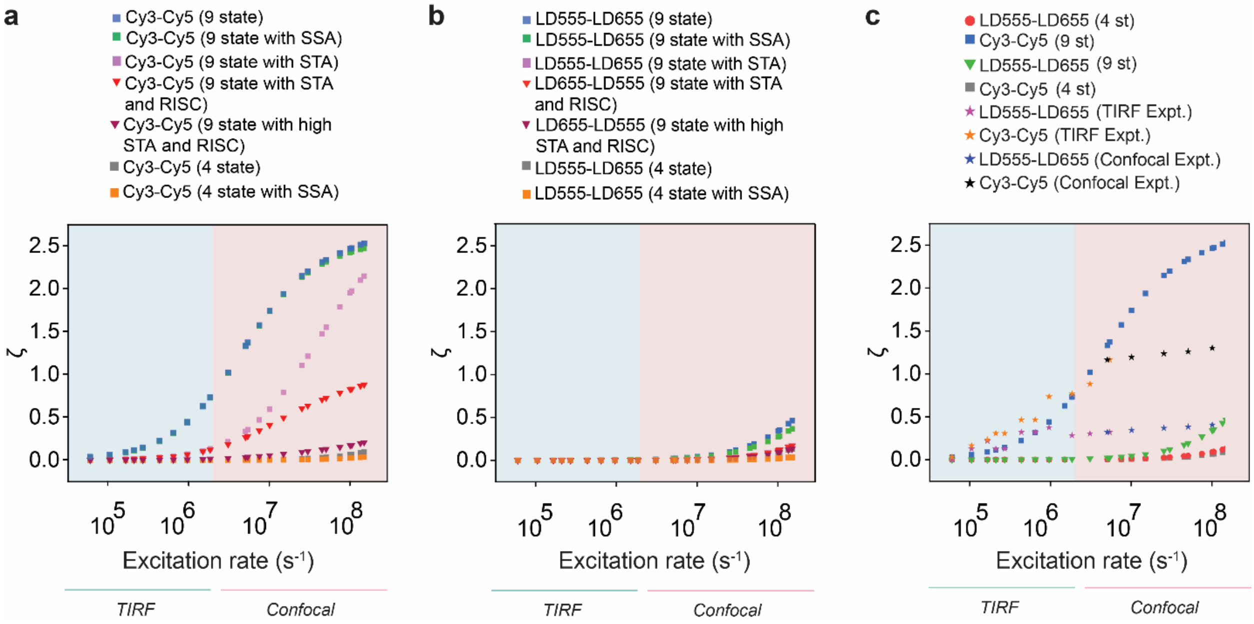

Supplementary Fig. 16. ζ correction parameters of Cy3-Cy5 and LD555-LD655 considering various excited state processes. (**a**,**b**) ζ correction in the presence and absence of alternative energy transfer pathways: singlet-singlet annihilation (SSA), single-triplet annihilation (STA) and reverse intersystem crossing (RISC). (a) Evaluated ζ correction for Cy3-Cy5 dye pairs (panel **a**) and LD555-LD655 dye pairs (panel **b**) where we assumed that $k_{SSA} = 5k_{ET}, k_{STA} = 10k_{ET}, k_{RISC} = 10^7\ (s^{-1})$ (rates are chosen to show how they are affecting the zeta correction for a specific distance) such that we include $S_1^D S_1^A \xrightarrow{k_{SSA}} S_0^D S_1^A$, $S_1^D T^A \xrightarrow{k_{STA}} S_0^D T^A$ for the SSA and STA investigation and $S_1^D T^A \xrightarrow{k_{STA}} S_0^D T^A$ with $S_1^D T^A \xrightarrow{k_{RISC}} S_0^D S_1^A$ for both STA and RISC investigation. For the high STA and RISC investigation (shown with maroon triangles), we used 10 times faster k_{STA} and k_{RISC} than the data provided with red triangles. (**c**) Variations of experimental and simulated ζ correction with excitation rates spanning from the TIRF to confocal excitation regimes.

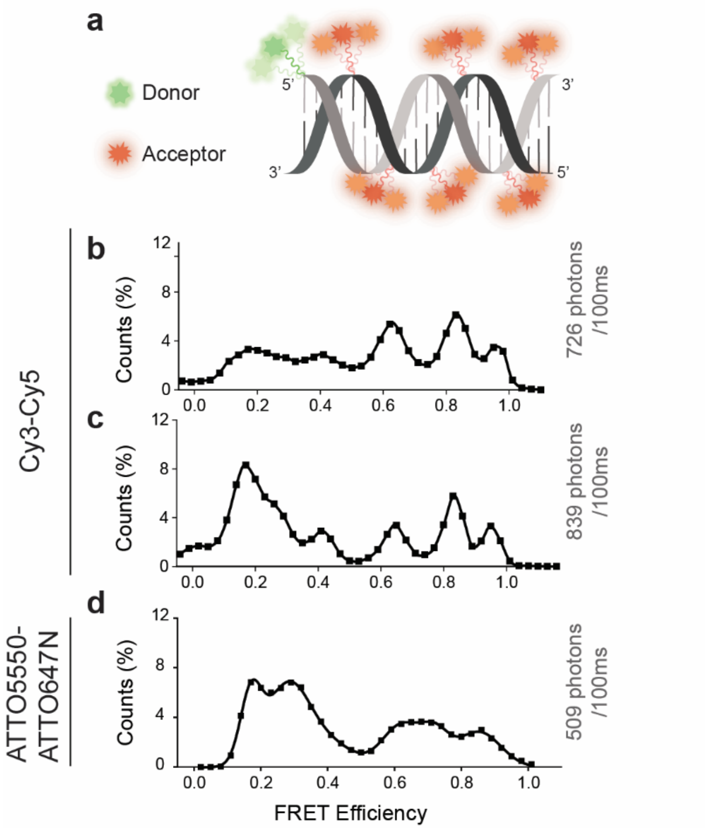

Supplementary Fig. 17. High spatial resolution FRET study of Cy3-Cy5 and ATTO550-ATTO647N. (**a**) Schematic diagram of a mixture of six DNA duplexes labeled with donor fluorophore at the 5'-terminus of one strand and acceptor fluorophore positioned at internal sites within the complementary strand separated by 5, 8, 11, 14, 17, and 20 nucleotides. (**b,c**) Population FRET histograms generated from experiments including all six DNA duplexes labeled with (**b**) Cy3-Cy5 in the presence of 1 mM ascorbic acid (AA) + 1 mM methyl viologen (MV) (ROX system)[4], (**c**) Cy3-Cy5 in the presence of 1 mM COT + 1 mM NBA + 1 mM Trolox (cocktail system)[5]. A single, empirically derived γ-correction parameter was used for FRET efficiency calculations. All these experiments were performed in deoxygenated imaging buffers at 100 ms time resolution and at 0.14 kW/cm^2. (**d**) Cumulative population FRET efficiency histograms of a cocktail of the six DNA duplexes labeled with ATTO550-ATTO647N pair in deoxygenated environments in the presence of 1 mM (AA + MV) at 100 ms time resolution at 0.11 kW/cm^2. A single, empirically derived γ-correction parameter was used for FRET efficiency calculations.

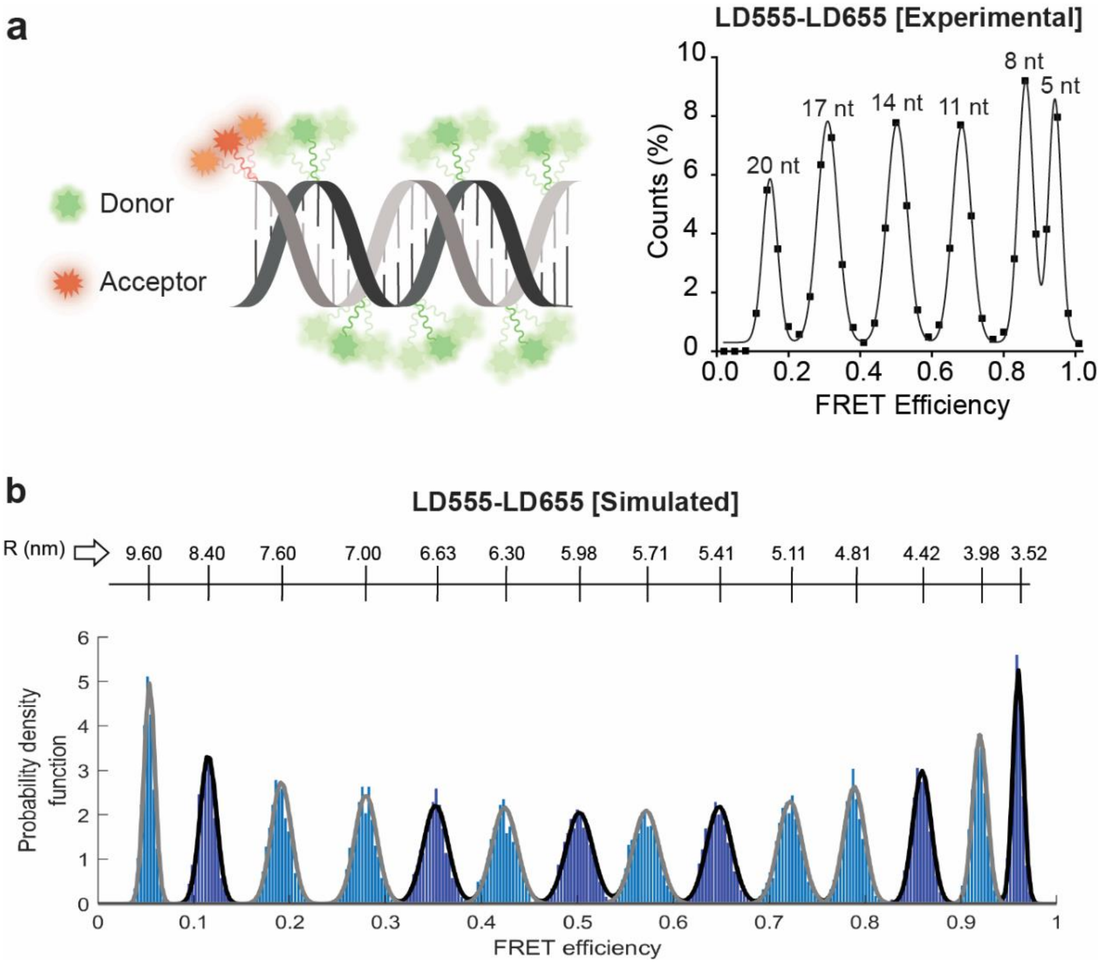

Supplementary Fig. 18. High spatial resolution FRET study of LD555-LD655. (**a**) Schematic diagram of a cocktail of six DNA duplexes labeled with an acceptor dye at the 5'-end of a DNA strand and a donor fluorophore at an internal position of its complementary DNA strand: 5, 8, 11, 14, 17, and 20 nucleotides apart from the acceptor fluorophore (left). FRET efficiency histogram of a cocktail of the six DNA duplexes labeled with LD555-LD655 in deoxygenated imaging buffers at 100 ms time resolution in the absence of any solution any additive (right). We used a single, empirically derived γ-correction parameter for FRET efficiency calculations. (**b**) Simulation of LD555-LD655 pair at different inter-dye distances, using photophysical parameters for the two dyes reported in **Supplementary Table 1**. The simulation was performed at 100 ms time resolution using 0.14 kW/cm^2 illumination intensity. The darker blue histograms with black fits correspond to FRET pairs that were also evaluated experimentally.

Supplementary Table 1. Photophysical parameters of FRET dyes.

Dyes	Quantum yield	k_{S_1} $(1/s)$	k_{em} $(1/s)$	k_{nr} $(1/s)$	$k_{nr}^{S_0}$ $(1/s)$	k_{TR} $(1/s)$	k_{ISC} $(1/s)$
**Cy3**[a]	0.21[b]	1.02×10^{9}[c]	0.21×10^{9}[d]	0.81×10^{9}[e]	0.8×10^{9}[e]	2.5×10^{4}[f]<br>2.2×10^{4}[g]	1×10^{7}[j]
**Cy5**[a]	0.31[b]	0.81×10^{9}[c]	0.25×10^{9}[d]	0.56×10^{9}[e]	0.55×10^{9}[e]	2.5×10^{4}[f]<br>2.2×10^{4}[g]	1×10^{7}[j]
**LD555**[a]	0.31[b]	0.76×10^{9}[c]	0.24×10^{9}[d]	0.52×10^{9}[e]	0.51×10^{9}[e]	1×10^{6}[f]	1×10^{7}[j]
**LD655**[a]	0.35[b]	0.59×10^{9}[c]	0.21×10^{9}[d]	0.38×10^{9}[e]	0.37×10^{9}[e]	5×10^{6}[f]	1×10^{7}[j]
**Cy3B**[a]	0.84[b]	0.35×10^{9}[c]	0.29×10^{9}[d]	0.56×10^{8}[e]	0.46×10^{8}[e]	1.4×10^{4}[h]<br>$(3.2 \pm 0.5) \times 10^{4}$[i]	1×10^{7}[j]
**Cy5B**	0.39[b]	0.49×10^{9}[c]	0.19×10^{9}[d]	0.30×10^{9}[e]	-	$(1.9 \pm 0.3) \times 10^{4}$[i]	-

[a]These dyes were individually labeled to DNA duplexes and were used for fluorescence quantum yield and fluorescence lifetime measurements. The labeling positions of the donor (Cy3, Cy3B, LD555) and acceptor (Cy5, LD655) dyes were at the same positions as depicted in **Fig. 2a**. [b]Fluorescence quantum yield data were measured in T50 buffer at room temperature. [c] k_{S_1} $(1/\tau_{S_1})$ data were calculated by taking a reciprocal of experimentally measured fluorescence lifetime data in T50 buffer at room temperature. [d]k_{em} (radiative rate constant) data were calculated by dividing experimentally measured fluorescence quantum yield by fluorescence lifetime. [e]k_{nr} (nonradiative rate constant) data were calculated by using the formula $(1 - Quantum\ yield)k_{S_1}$[6], the nonradiative relaxation rate from S_1 to S_0 is denoted by $k_{nr}^{S_0} = k_{nr} - k_{ISC}$. [f]k_{TR} (triplet relaxation rate constant) data were calculated by taking a reciprocal of measured triplet state lifetime of free dyes in deaerated acetonitrile solvent using transient absorption spectroscopy and intramolecular triplet sensitization[7,8]. [g]k_{TR} values used in our studies in aqueous buffers under enzymatically deoxygenated conditions derived from simulations, which closely recapitulate experimental FRET efficiency values. In aqueous buffers modestly faster triplet relaxation rates due to both the solvent and the presence of residual levels of molecular oxygen, which we estimate to be approximately 10 µM at steady state. [h]k_{TR} data was calculated by taking a reciprocal of literature reported triplet state lifetime of free dye in argon-equilibrated PBS buffer, pH 7.4 in the presence of 50 mM potassium iodide[9]; this value was used for the simulation of Cy3B emission data in **Supplementary Fig.11** and was in reasonable agreement with our measured [i]k_{TR} value obtained through fluorescence correlation spectroscopy experiments in deoxygenated T50 imaging buffer (**Supplementary Fig. 8**). [j]k_{ISC} (intersystem crossing rate constant) values were optimized through simulations (**Supplementary Fig. 12a,c**), which agreed well with the literature[10–13]. [k]k_{TR} value of free Cy5B dye obtained through fluorescence correlation spectroscopy experiments in deoxygenated T50 imaging buffer (**Supplementary Fig. 8**). The fluorescence quantum yield and fluorescence lifetime measurements of Cy5B were made using free dyes in T50 buffer. The chemical structures of all the dyes are provided in **Supplementary Fig. 1**.

Supplementary Table 2. Förster radius for the dye pairs used in **Fig. 2**.

Dye pairs	Förster radius
**Cy3-Cy5**	5.69 nm
**LD555-LD655**	6.07 nm

The Förster radius values were calculated as described previously[14], using experimentally obtained quantum yields in **Supplementary Table 1**, κ^2 of 2/3, refractive index of 1.333, excitation spectra of free fluorophore in water, and emission spectra measured for fluorophore labelled to DNA duplexes.

Supplementary Table 3. Description of theoretical FRET efficiency for 4 and 9 state models.

Formula	Rate description
$E_{theory}^{4\,st} = \frac{k_{ET}^{D}}{(k_{ET}^{D} + k_{S_1}^{D})}$	***4 state model***<br>$k_{S_1}^{D} = k_{em}^{D} + k_{nr}^{S_0^D}$
$E_{theory}^{9\,st} = \frac{k_{ET}^{D}}{(k_{ET}^{D} + k_{S_1}^{D})}$	***9 state model***<br>$k_{S_1}^{D} = k_{em}^{D} + k_{nr}^{S_0^D} + k_{ISC}^{D}$

Supplementary Table 4. Description of simulated and experimental FRET efficiencies.

Notation	Formula	Additional formula	Description
E_{sim}	$E_{sim} = \frac{I_A^{sim}}{I_A^{sim} + \gamma^{sim} I_D^{sim}}$	$\gamma^{sim} = \frac{\lvert < I_A^{sim,FRET} > - < I_A^{sim,No\ FRET} > \rvert}{\lvert < I_D^{sim,FRET} > - < I_D^{sim,No\ FRET} > \rvert}$	Simulations are carried out based on the photophysical model with the experimentally provided model parameters.
E_{exp}	$E_{exp} = \frac{I_A^{exp}}{I_A^{exp} + \gamma^{exp} I_D^{exp}}$	$\gamma^{exp} = \frac{\lvert < I_A^{exp,FRET} > - < I_A^{exp,No\ FRET} > \rvert}{\lvert < I_D^{exp,FRET} > - < I_D^{exp,No\ FRET} > \rvert}$	Experiments are carried out as explained in the “smFRET imaging data analysis” section.

Supplementary Table 5. Description of theoretical FRET efficiencies from $E^{4\,st}_{theory}$.

Notation	Formula	Additional formula	Description
$\boldsymbol{E^{4\,st}_{theory}}$	$E^{4\,st}_{theory} = \frac{k^D_{ET}}{(k^D_{ET} + k^D_{S_1})}$	k^D_{ET} is computationally estimated based on the experimental data.	$k^D_{ET}, k^D_{S_1}$ are known from experiments.
$E_{4\,st}$	$E_{4\,st} = \frac{\rho_A}{\rho_A + (\frac{\eta_A \varphi_A}{\eta_D \varphi_D})\rho_D}$	$\rho_D = \eta_D k^D_{S_1} \varphi_D (S^D_1 S^A_0 + S^D_1 S^A_1)$,<br>$\rho_A = \eta_A k^A_{S_1} \varphi_A (S^D_0 S^A_1 + S^D_1 S^A_1)$.	$S^D_1 S^A_0, S^D_1 S^A_1, S^D_0 S^A_1, S^D_1 S^A_1$ represent steady state population fractions for excited donor and acceptor populations for 4 state photophysical model.
$\gamma^{4\,st}_{theory}$	$\gamma^{4\,st}_{theory} = \frac{\rho_A - \rho^{No\ FRET}_A}{\rho^{No\ FRET}_D - \rho_D}$	$\rho_D = \eta_D k^D_{S_1} \varphi_D (S^D_1 S^A_0 + S^D_1 S^A_1)$,<br>$\rho_A = \eta_A k^A_{S_1} \varphi_A (S^D_0 S^A_1 + S^D_1 S^A_1)$,<br>$\rho^{No\ FRET}_A = 0\ (with\ no\ direct\ excitation)$,<br>$\rho^{No\ FRET}_D$ is obtained by setting $k^D_{ET} = 0$.	$\gamma^{4\,st}_{theory} = \frac{\eta_A k^D_{ex}}{\eta_D k^D_{em}} \varphi_A + \gamma$ where $\gamma = \frac{\eta_A \varphi_A}{\eta_D \varphi_D}$.

Supplementary Table 6. Description of theoretical FRET efficiencies from $E^{9\,st}_{theory}$.

Notation	Formula	Additional formula	Description
$E^{9\,st}_{theory}$	$E^{9\,st}_{theory} = \frac{k^D_{ET}}{(k^D_{ET} + k^D_{S_1})}$	k^D_{ISC} is computationally estimated based on the experimental data.	$k^D_{ET}, k^D_{S_1}$ are known from experiments.
$E_{9\,st}$	$E_{9\,st} = \frac{\rho_A}{\rho_A + (\frac{\eta_A \varphi_A}{\eta_D \varphi_D})\rho_D}$	$\rho_D = \eta_D k^D_{S_1} \varphi_D (S^D_1 S^A_0 + S^D_1 S^A_1 + S^D_1 T^A)$, $\rho_A = \eta_A k^A_{S_1} \varphi_A (S^D_0 S^A_1 + S^D_1 S^A_1 + T^D S^A_1)$.	$S^D_1 S^A_0, S^D_1 S^A_1, S^D_1 T^A, S^D_0 S^A_1, S^D_1 S^A_1, T^D S^A_1$ represent steady state population fractions for excited donor and acceptor populations for 9 state photophysical model.
E_{true}	$E_{true} = \frac{1}{\frac{1}{E_{exp}} - \zeta}$	ζ is provided in "ζ correction protocol for FRET efficiency" section.	-
$\gamma^{9\,st}_{theory}$	$\gamma^{9\,st}_{theory} = \frac{\rho_A - \rho^{No\,FRET}_A}{\rho^{No\,FRET}_D - \rho_D}$	$\rho_D = \eta_D k^D_{S_1} \varphi_D (S^D_1 S^A_0 + S^D_1 S^A_1 + S^D_1 T^A)$, $\rho_A = \eta_A k^A_{S_1} \varphi_A (S^D_0 S^A_1 + S^D_1 S^A_1 + T^D S^A_1)$, $\rho^{No\,FRET}_A = 0$, (with no direct excitation) $\rho^{No\,FRET}_D$ is obtained by setting $k^D_{ET} = 0$.	$\gamma^{9\,st}_{theory} = \frac{\eta_A k^D_{ex}}{\eta_D k^D_{em}} \varphi_A + \frac{\eta_A k^D_{ex} k^D_{ISC}}{\eta_D k^D_{em} k^D_{TR}} \varphi_A + \gamma$ where $\gamma = \frac{\eta_A \varphi_A}{\eta_D \varphi_D}$.

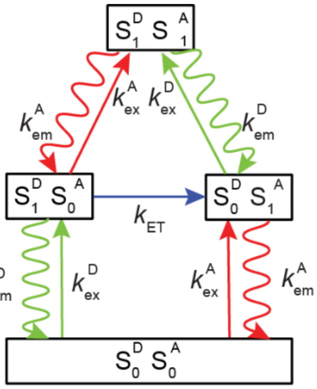

Supplementary Scheme 1: A 4-state photophysical model.

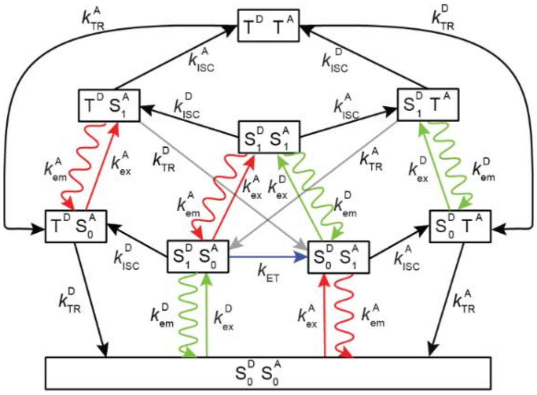

Supplementary Scheme 2: A 9-state photophysical model.

References

1. Roy, R., Hohng, S. & Ha, T. A practical guide to single-molecule FRET. *Nat. Methods* **5,** 507–516 (2008).

2. Hellenkamp, B. *et al.* Precision and accuracy of single-molecule FRET measurements-a multi-laboratory benchmark study. *Nat. Methods* **15,** 669–676 (2018).

3. Hildebrandt, L. L., Preus, S. & Birkedal, V. Quantitative single molecule FRET efficiencies using TIRF microscopy. *Faraday Discuss* **184,** 131–142 (2015).

4. Vogelsang, J., Cordes, T., Forthmann, C., Steinhauer, C. & Tinnefeld, P. Controlling the fluorescence of ordinary oxazine dyes for single-molecule switching and superresolution microscopy. *Proc. Natl. Acad. Sci. USA* **106,** 8107–8112 (2009).

5. Dave, R., Terry, D. S., Munro, J. B. & Blanchard, S. C. Mitigating unwanted photophysical processes for improved single-molecule fluorescence imaging. *Biophys. J.* **96,** 2371–2381 (2009).

6. Lakowicz, J. R. *Principles of Fluorescence Spectroscopy*. (Springer US, 2006). doi:10.1007/978-0-387-46312-4

7. Zheng, Q. *et al.* Electronic tuning of self-healing fluorophores for live-cell and single-molecule imaging. *Chem. Sci.* **8,** 755–762 (2017).

8. Pati, A. K. *et al.* Tuning the Baird aromatic triplet-state energy of cyclooctatetraene to maximize the self-healing mechanism in organic fluorophores. *Proc. Natl. Acad. Sci. USA* **117,** 24305–24315 (2020).

9. Glembockyte, V. & Cosa, G. Redox-Based Photostabilizing Agents in Fluorescence Imaging: The Hidden Role of Intersystem Crossing in Geminate Radical Ion Pairs. *J. Am. Chem. Soc.* **139,** 13227–13233 (2017).

10. Chibisov, A. K., Zakharova, G. V. & Görner, H. Effects of substituents in the polymethine chain on the photoprocesses in indodicarbocyanine dyes. *J. Chem. Soc., Faraday Trans.* **92,** 4917–4925 (1996).

11. Eggeling, C. *et al.* Analysis of photobleaching in single-molecule multicolor excitation and Förster resonance energy transfer measurements. *J. Phys. Chem. A* **110,** 2979–2995 (2006).

12. Song, L., Varma, C. A., Verhoeven, J. W. & Tanke, H. J. Influence of the triplet excited state on the photobleaching kinetics of fluorescein in microscopy. *Biophys. J.* **70,** 2959–2968 (1996).

13. Sakhapov, D., Gregor, I., Karedla, N. & Enderlein, J. Measuring Photophysical Transition Rates with Fluorescence Correlation Spectroscopy and Antibunching. *J. Phys. Chem. Lett.* **13,** 4823–4830 (2022).

14. Girodat, D., Pati, A. K., Terry, D. S., Blanchard, S. C. & Sanbonmatsu, K. Y. Quantitative comparison between sub-millisecond time resolution single-molecule FRET measurements and 10-second molecular simulations of a biosensor protein. *PLoS Comput. Biol.* **16,** e1008293 (2020).
